# BRCA1 Antibodies Matter

**DOI:** 10.7150/ijbs.63115

**Published:** 2021-07-25

**Authors:** Jing Yang, Leilei Qi, Huai-Chin Chiang, Bin Yuan, Rong Li, Yanfen Hu

**Affiliations:** 1 Department of Anatomy & Cell Biology, School of Medicine & Health Sciences, The George Washington University, Washington, DC, USA; 2 Department of Biochemistry & Molecular Medicine, School of Medicine & Health Sciences, The George Washington University, Washington, DC, USA

**Keywords:** BRCA1, Antibody validation, Western blot, Immunoprecipitation, Chromatin Immunoprecipitation, Immunofluorescence

## Abstract

*Breast cancer susceptibility gene 1* (*BRCA1*) encodes a tumor suppressor that is frequently mutated in familial breast and ovarian cancer patients. BRCA1 functions in multiple important cellular processes including DNA damage repair, cell cycle checkpoint activation, protein ubiquitination, chromatin remodeling, transcriptional regulation, as well as R-loop formation and apoptosis. A large number of BRCA1 antibodies have been generated and become commercially available over the past three decades, however, many commercial antibodies are poorly characterized and, when widely used, led to unreliable data. In search of reliable and specific BRCA1 antibodies (Abs), particularly antibodies recognizing mouse BRCA1, we performed a rigorous validation of a number of commercially available anti-BRCA1 antibodies, using proper controls in a panel of validation applications, including Western blot (WB), immunoprecipitation (IP), immunoprecipitation-mass spectrometry (IP-MS), chromatin immunoprecipitation (ChIP) and immunofluorescence (IF). Furthermore, we assessed the specificity of these antibodies to detect mouse BRCA1 protein through the use of testis tissue and mouse embryonic fibroblasts (MEFs) from Brca1^+/+^ and Brca1^Δ11/Δ11^ mice. We find that Ab1, D-9, 07-434 (for recognizing human BRCA1) and 287.17, 440621, BR-64 (for recognizing mouse BRCA1) are specific with high quality performance in the indicated assays. We share these results here with the goal of helping the community combat the common challenges associated with anti-BRCA1 antibody specificity and reproducibility and, hopefully, better understanding BRCA1 functions at cellular and tissue levels.

## Introduction

Antibodies (Abs) are a key resource and one of most frequently used tools in biomedical research 1, 2. There are more than one million commercially available antibodies on offer 1, however, rigorous antibody validation efforts tend to fall behind antibody generation. Utilization of poor quality antibodies has been estimated to cost $350 million in the United States and $800 million globally each year as a result of unreliable experiments 3.

Germline mutations in tumor suppressor gene *BRCA1* are highly penetrant for the increased risk of familial breast and ovarian cancer occurrence 4-6. A wealth of molecular functions of BRCA1 has been identified since the gene was identified in 1994. Human BRCA1 is a large protein with multiple functional domains and forms several distinct complexes that are involved in many important cellular activities such as DNA damage repair, cell cycle checkpoint control, protein ubiquitination, chromatin remodeling, transcriptional regulation, as well as R-loop formation 7-15. Over the years, a large number of BRCA1 antibodies have been generated and become commercially available. It is relatively common in the BRCA1 field that many antibodies were initially confirmed based on simple Western blotting analysis, but eventually used in various applications without rigorous antibody characterization. For example, published BRCA1 ChIP and ChIP-seq results in literature rarely overlapped or are hard to reproduce 16, 17. Although this inconsistency could be attributed to difference in cell lines or other potential experimental contextual differences, it is equally possible that many BRCA1 antibodies used in studies recognize strong nonspecific bands on Western blotting, or their specificity is poorly characterized, therefore could introduce high noise background in ChIP. The problem is hard to tackle because BRCA1 is not known to bind to DNA in a sequence-specific manner and could bind anywhere through protein-protein interactions. Currently there are no simple alternative ways to verify BRCA1 target regions identified by ChIP analysis, making it especially important that BRCA1 antibodies are truly specific. Of note, most of our current knowledge of BRCA1 derived from studies using human cancer cell lines. Studies using BRCA1 mouse models have not been fully explored, especially at molecular level using mouse derived cells. For example, after 30 years of extensive studies, the role of mouse BRCA1 and even its expression pattern in cell and tissue types are still unclear. This is partly due to lacking well characterized antibodies recognizing mouse BRCA1. Investigators sometimes performed Western blotting with BRCA1 antibodies that are unverified or no detailed information provided 18-20, or by using genomic DNA and/or mRNA analyses as indicative of BRCA1 deletion/depletion in transgenic animal studies 21-25. In our studies of BRCA1 using mouse cells, we found that several commercial BRCA1 antibodies did recognize a band with expected size on Western blot, however, the intensity of the presumed “BRCA1” band did not change when endogenous BRCA1 was knocked down using siRNA. While we confirmed the knockdown of BRCA1 at mRNA level by RT-PCR, a caveat of this approach is that we could not rule out the possibility that BRCA1 mRNA and protein level may not always be in sync. We therefore decided to carry out a thorough rigorous characterization of all commercial BRCA1 antibodies in terms of their specificity in human and mouse cells in several common applications.

The International Working Group on Antibody Validation (IWGAV) in 2016 proposed five conceptual pillars to guide antibody validation in specific research applications: (1) genetic strategies: measure the relevant signal in control cells or tissues in which the target gene has been knocked down or knocked out (KO); (2) orthogonal strategies: use an antibody-independent method for quantification across multitudes of samples and then examine the correlation between the antibody-based and antibody-independent quantifications; (3) independent antibody strategies: use two or more independent antibodies that recognize different epitopes on the target protein and confirm specificity via comparative and quantitative analyses; (4) expression of tagged proteins: modify the endogenous target gene to add sequences for an affinity tag or a fluorescent protein. The signal from the tagged protein can be correlated with detection through antibody-based methods; (5) IP-MS: isolate a protein from a solution through binding with a target-specific antibody, followed by mass-spectrometry (MS) analysis to identify proteins that interact with the purified antibody 2, 26. The most frequent mistake made with antibodies used in the scientific community is that antibody specificity is not comprehensively experimentally confirmed before use 27.

Here, we present the first comprehensive study of BRCA1 antibody validation for their ability to detect human and mouse BRCA1 by a number of assays, including Western blot, IF, IP, ChIP-qPCR, IP-MS analyses. We hope that these results could serve as a useful reference for other labs working on BRCA1. Specifically, we identified several antibodies that recognize mouse BRCA1 by performing multiple antibody-based applications with proper controls. We hope that the work presented in this manuscript will not only be valuable to the BRCA1 community, but also be meaningful for setting up a standard antibody validation strategy in general.

## Materials and methods

### Cell Culture

Human triple-negative breast cancer cell line MDA-MB-468 and osteosarcoma cell line U2OS were purchased from ATCC and cultured in high glucose DMEM (Thermo Fisher Scientific; 11965) supplemented with 10% fetal bovine serum (FBS), 100 μg ml^-1^ penicillin and 100 μg ml^-1^ streptomycin (Thermo Fisher Scientific; 15140122). Non-targeting siRNA control pool (Dharmacon; D-001810-10) was purchased as a negative control. Human BRCA1 siRNAs were synthesized from Sigma-Aldrich. The target sequences of BRCA1 siRNA are follows: GAAGCCAGCTCAAGCAATA (DO3); GCAGATAGTTCTACCAGTA (DO4); AAGGTTTCAAAGCGCCAGTCA (CR); ACCATACAGCTTCATAAATAA (NAR3). Lipofectamine RNAiMAX transfection reagent (Thermo Fisher Scientific; 13778150) was used to transfect BRCA1 siRNA or control siRNA into cells at final concentration of 25 nM following manufacturer's instructions. Three days later, BRCA1 protein levels were analyzed using immunoblots to determine the efficiency of knockdown.

The inducible stable Cas9 Hela cell lines, control (CTT20) and BRCA1 KO (A9.2 and A10.2), were generated by Dr. Iain M.Cheeseman's lab at Massachusetts Institute of Technology 28, 29. Cells were cultured in high glucose DMEM supplemented with 10% tetracycline-free FBS, penicillin/streptomycin and 2 mM L-glutamine (Gibco). To induce Cas9 protein expression, cells were cultured in culture medium supplemented with 100 ng/ml doxycycline hyclate (Dox; Sigma) for 72 h, before collecting cells for immunoblots.

### Antibodies

### Western blot

Cells were pelleted by centrifugation (1,500 rpm, 5 min, at 4 °C) and washed in cold phosphate buffered saline (PBS). Cell pellets were lysed in RIPA Lysis and Extraction Buffer. Mouse testis lysate was prepared by homogenization in T-PER™ Tissue Protein Extraction Reagent (Thermo Fisher Scientific; 78510). Tissue and cell debris were removed by centrifugation (13,000 rpm, 10 min, at 4 °C). Protein concentrations were determined by using Pierce BCA Protein Assay Kits (Pierce; Cat: #23225). For Western blot analysis of hBRCA1 and mBRCA1 proteins, 20-30 μg of each lysate was separated by 6% Novex Tris-Glycine gel (Invitrogen). Membranes were probed with anti-BRCA1 antibodies overnight at 4 °C with gentle shaking. And the corresponding horseradish peroxidase (HRP) conjugated secondary antibodies were used. Proteins were visualized using ECL SuperSignalTM West Pico PLUS Chemiluminescent Substrate (Thermo Fisher, Cat. #34580).

### Immunoprecipitation (IP)

The plasmid pCMV3-C-Flag human BRCA1 was purchased from Sino Biological Inc. (Beijing, China). 293T cells were transfected with Flag-BRCA1, and cell lysates were collected 48h after transfection for immunoprecipitation. U2OS/3xFLAG-APEX2-fused BRCA1 cell line was generated by Dr. Chunaram Choudhary's group at University of Copenhagen 30. Briefly, 293T/Flag-BRCA1, U2OS/3xFLAG-APEX2-fused BRCA1 and MDA-MB-468 cells were lysed in ice-cold lysis buffer (10 mM Tris-HCl [pH 7.5], 150 mM NaCl, 0.025% SDS, 0.1% sodium deoxycholate, 0.5% Nonidet P-40, 5 mM EDTA) supplemented with protease inhibitor and phosphatase inhibitor cocktails, and then cleared by centrifugation at 2,000 rpm for 10 minutes. The lysate was rotated with indicated BRCA1 antibodies at 4 °C overnight. Protein A/G agarose beads (Thermo Fisher Scientific; 20423) were then added, and rotated for another 2 h at 4 °C. After vigorous washing, bound proteins were analyzed by immunoblotting using the indicated antibodies.

### ChIP-qPCR

ChIP was performed according to manufacturer's instructions for the ChIP-IT High Sensitivity Kit (Active Motif; 53040). Briefly, ∼10^7^ U2OS or Hela cells were cross-linked with 1% formaldehyde for 10 min at 37 °C, and formaldehyde was quenched by adding glycine to a final concentration of 0.125 M for 5 min. Subsequently, lysates were sonicated using a Q800R sonicator (QSonica). Sonicated solution was diluted and precleared with protein A/G agarose beads. Cleared chromatin solutions were immunoprecipitated with indicated BRCA1 antibodies and incubated overnight at 4°C. The normal mouse and rabbit IgG (Vector Laboratories; I-2000-1 and I-1000-5) were used as negative controls. Subsequently, 50μl of protein A/G agarose beads were added to each ChIP reaction and incubated for 2 hours at 4°C. The chromatin was eluted, followed by reverse crosslink and DNA purification. The Input and ChIP DNA was used in qPCR with specific PCR primers using SYBR Green Supermix (Bio-Rad) and the ABI 7900HT Fast Real-Time PCR System. Primers for each gene are follows:

BRCA1 (F: 5'-CCATCTGTCAGCTTCGGAAA -3' and R: 5'-TGCTCTGGGTAAAGGTAGTAGA-3')

ATRIP (F: 5'-GGACTTCACTGCCGACGAC -3' and R: 5'-CGGTTGACAACTCCCTCCG-3')

EXO1 (F: 5'-TCAACATCAGCCTCCAGAAC-3' and R: 5'-TCGGAAGTTGGGAGTGTTTAC-3')

MAD2L1 (F: 5'-CTTTCTCTCAGCCTTCCTGTG-3' and R: 5'-CGACCAGAAGACACATCCTAAC-3')

PPM1D (F: 5'-CAGCAGGCCGCATTAAGA-3' and R: 5'-TCGGCAGTTGTTGATCCTTT-3')

### Laser Micro-irradiation and Immunofluorescence (IF)

U2OS cells or MEFs cells were seeded in 8-well chamber slides and micro-irradiated 20 hours later when cells reached 80% confluency. Micro-irradiation was performed using an MMI Cell Cut laser microdissection system consisting of a 390 nm ND-YAG laser that is coupled to the optical path of the microscope. The IF staining was performed as described by us previously 31. Briefly, cells were fixed 30 minutes after laser micro-irradiation with either a 3% Paraformaldehyde/2% Sucrose solution in Phosphate Buffered Saline (PBS/pH 7.4) for 15 minutes at room temperature or 70% Methanol/30% Acetone for 15 minutes at -20C. After fixation, cells were permeabilized in 0.5% Triton X-100 buffer (10 mM PIPES, pH 6.8, 50 mM NaCl, 1 mM EDTA, 3 mM MgCl2, 300 mM sucrose, and 0.5% Triton X-100) with 10% FBS for 5 minutes on ice. After blocking for 40 minutes followed by incubating with primary antibody (anti-Mouse Brca1 and anti-Rabbit γH2AX or anti-Rabbit Brca1 and anti-Mouse γH2AX) overnight in a cold room and secondary antibody (anti-Rabbit, Alexa Fluor 488 and anti-Mouse Alexa Fluor 546) for 2 hours at room temperature, slides were mounted with a coverslip using Vectashield with DAPI (Vector laboratories) and imaging was done using Zeiss 710 Confocal microscope.

### Mice

All animal related experiments were approved by The George Washington University (GWU) Institutional Animal Care and Use Committee. P53^+/-^Brca1^Co/Co^MMTV-Cre was obtained from Dr. Priscilla Furth's lab at Georgetown University 32. Whole body p53^+/-^ ; Brca1^+/Δ11^ mice derived from p53^+/-^; Brca1^Co/Co^; MMTV-Cre breeding due to MMTV-Cre leakage in oocytes were used for inbreeding to obtain p53^+/-^; Brca1^Δ11/Δ11^ and p53^+/-^; Brca1^+/+^ littermates.

### Generation and immortalization of MEF cells

Mouse embryos (13.5-14.5 days postcoitum) generated from intercrosses of p53^+/-^ Brca1^+/Δ11^ mice were dissected and internal organs removed described earlier 33. Briefly, dissociation was performed by mincing embryos with 2 ml Trypsin/EDTA followed by 20 min incubation at 37°C. Pipet the embryos in Trypsin/EDTA vigorously, and incubate for another 10 min at 37°C. After centrifugation, cell pellet was resuspended in 100 mm cell culture dish. The immortalized p53^+/-^; Brca1^+/+^ and p53^+/-^; Brca1^Δ11/Δ11^ cells were generated by the regular 3T3 protocol 34.

### Mass spectrometry (MS)

The nuclear extract of p53^+/-^; Brca1^+/+^ MEFs was used for BRCA1 immunoprecipitation by Nuclear Extraction Kit (Abcam; ab113474) according to the manufacturer's instructions. BRCA1 IP was performed with indicated BRCA1 antibodies using Pierce Classic IP Kit (Thermo Fisher Scientific; 26146) according to the manufacturer's instructions. For peptide analyses, samples were separated by gel electrophoresis after immunoprecipitation, corresponding areas were cut out, in-gel digested and analyzed by nano LC-MS/MS by Creative Proteomics.

## Results

### Analysis of BRCA1 antibodies by immunoprecipitation in human cell lines

**Figure 1:**
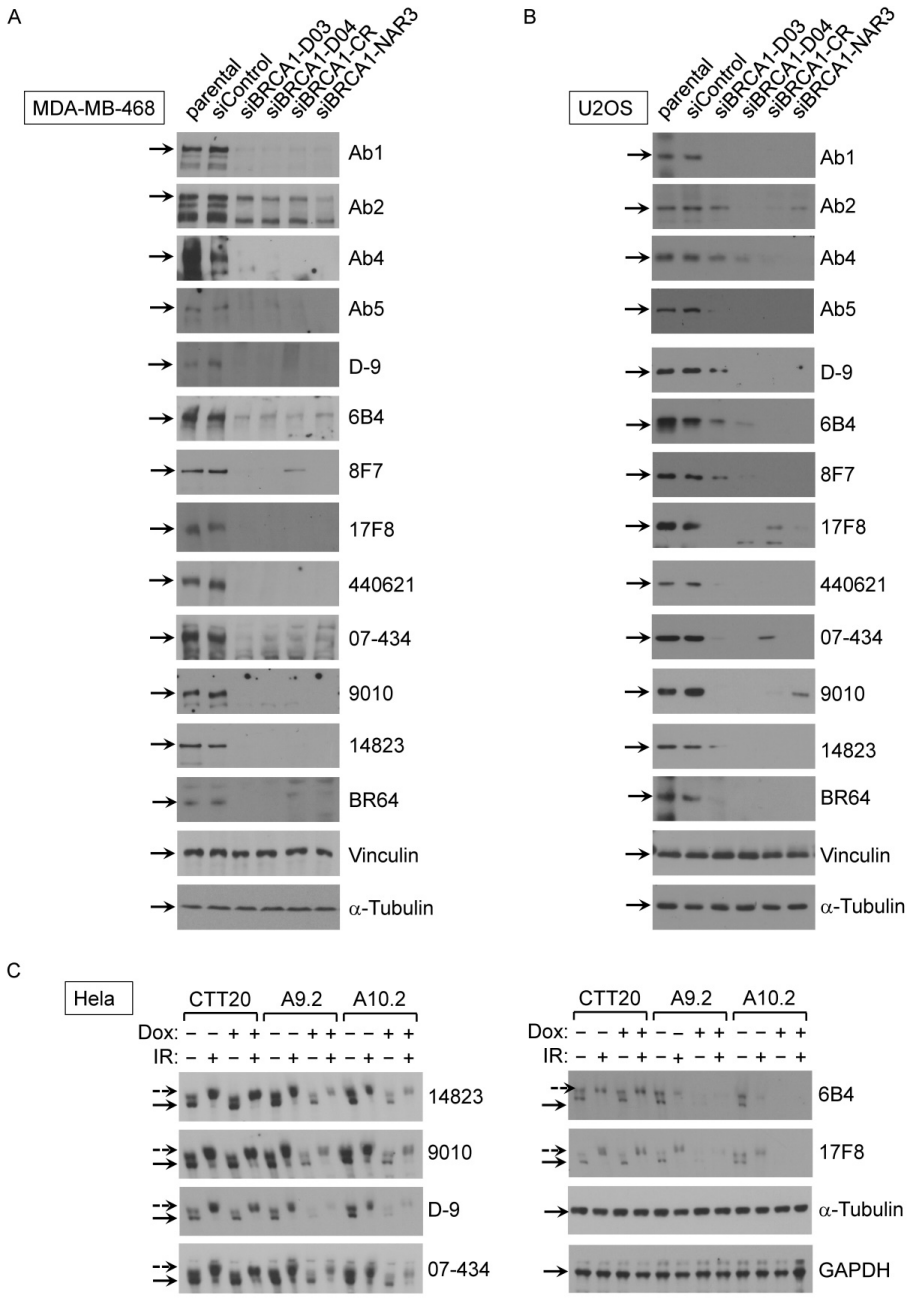
** Validation of BRCA1 antibodies in western blot application with human cancer cell lines. (A)** BRCA1 Abs test with MDA-MB-468 cells with/without siRNA transfection.** (B)** BRCA1 Ab test with U2OS cells with/without BRCA1 siRNA transfection.** (C)** BRCA1 Ab test with Hela inducible BRCA1 KO cell lines with/without IR.

Although antibody performance is assay-dependent, Western blotting remains the most commonly performed assay for assuring antibody specificity 35. As a first step, we sought to validate 23 commercial BRCA1 antibodies on their recognizing human BRCA1 protein in Western blot assay. Crude lysates of MDA-MB-468 and U2OS cells including parental, control siRNA and 4 different BRCA1 siRNA lines were used in initial Western blot. Tubulin and vinculin were served as two independent loading controls of cell lysates. After screening all 23 antibodies, we found that 13 antibodies could detect full-length human BRCA1 protein (Fig. 1A-B), as judged by knockdown controls. The other 10 antibodies either recognized a band at correct size but the intensity of the band did not decrease in siRNA samples or could not detect signal at the full length BRCA1 position. Among these 13 validated antibodies, 6 were further confirmed using an inducible BRCA1 knockout HeLa cell system (Fig. 1C). In this HeLa cell derived system, the control line CTT20 expresses endogenous BRCA1 because tetracycline-inducible Cas9 is silent. Two inducible BRCA1 knockout (KO) cell lines, A9.2 and A10.2 28, 29, have significantly reduced BRCA1 expression when Cas9 expression is activated in the presence of Doxycycline. BRCA1 signal was not completely abolished in KO cell lines A9.2 and A10.2, most likely because induced knockout mediated by Cas9 expression could not reach 100% efficiency. Both knockdown and knockout results show that these antibodies recognize human BRCA1 specifically. Consistent with previous findings, BRCA1 phosphorylation occurred upon gamma irradiation as indicated.

**Figure 2:**
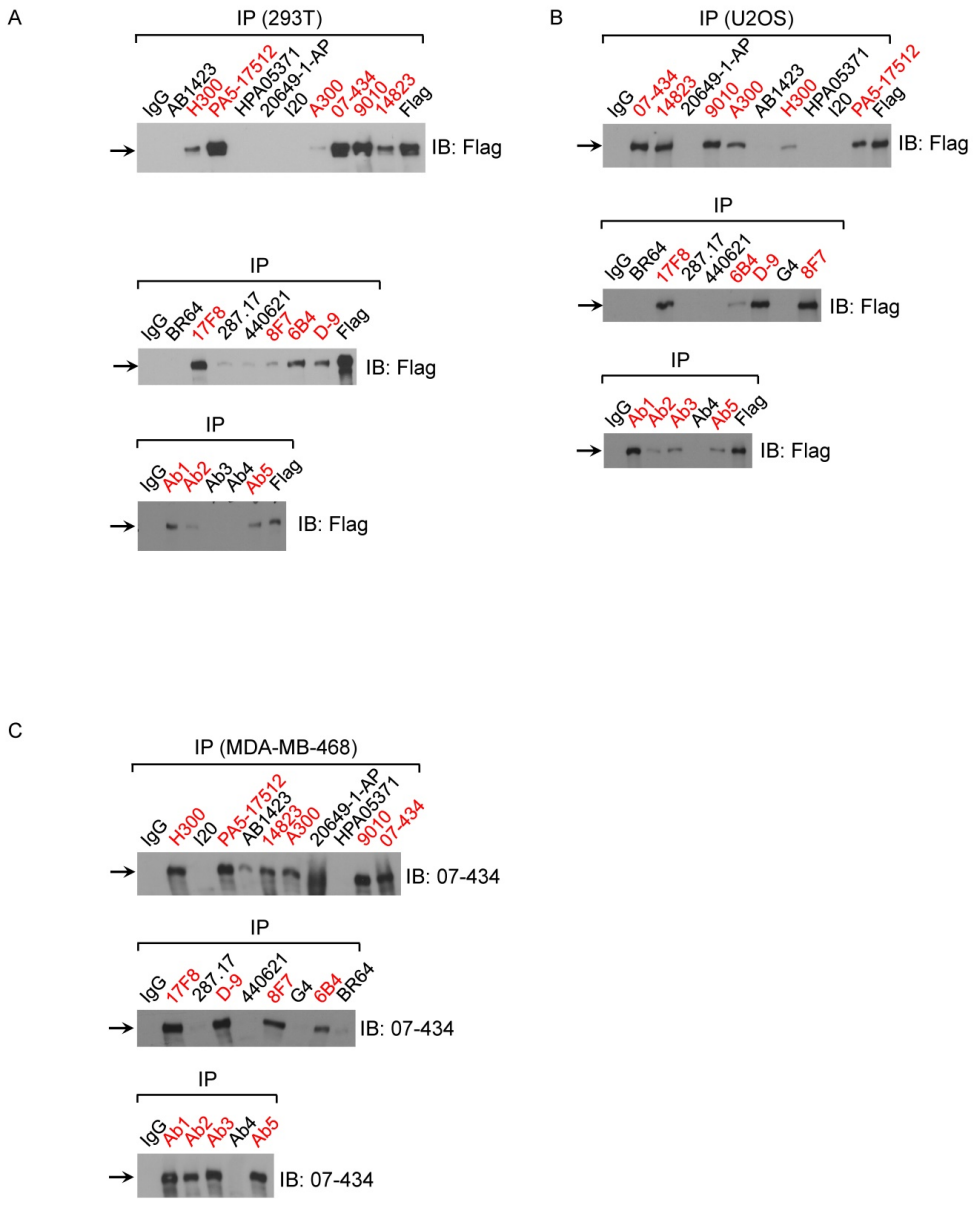
** Analysis of BRCA1 antibodies by immunoprecipitation in human cell lines. (A)** BRCA1 IPs with 293T/3XFlag-hBRCA1 stably transfected cell lysate. **(B)** BRCA1 IPs with U2OS/3xFLAG-APEX2-fused BRCA1 cell lysate. **(C)** BRCA1 IPs with MDA-MB-468 cell lysate.

Next, we examined which commercial BRCA1 antibodies could immunoprecipitate (IP) exogenous and endogenous BRCA1 protein using following cell lines: 293T/Flag-BRCA1 (Fig. 2A), U2OS/3xFLAG-APEX2-fused BRCA1 (Fig. 2B) and MDA-MB-468 (Fig. 2C). First, using lysates from 293T/Flag-BRCA1 and U2OS/3xFLAG-APEX2-fused BRCA1 30, we showed that a number of antibodies (D-9, H300, #9010, #14823, # PA5-17512, 6B4, 8F7, 17F8, A300, 07-434, Ab1, Ab2, Ab5) were able to IP human full-length BRCA1 protein at the correct size confirmed by immunoblotting (IB) with anti-Flag antibody. We further tested IP with lysates from MDA-MB-468 cells and got similar results using anti-BRCA1 antibody (07-434) in immunoblotting. Taken together, these results show that multiple anti-BRCA1 antibodies can immunoprecipitate both endogenous and exogenously expressed full-length human BRCA1 protein. Curiously, H300 and PA5-17512 could IP full length human BRCA1 efficiently in all three cell lines, but ambiguous in Western blot, as they both detected a signal at full length BRCA1 position, but the signal did not decrease when BRCA1 was knocked down. It is possible that these two antibodies recognize BRCA1 in its native form, but not denatured BRCA1.

### Analysis of BRCA1 antibodies by immunofluorescence in human U2OS cells

**Figure 3:**
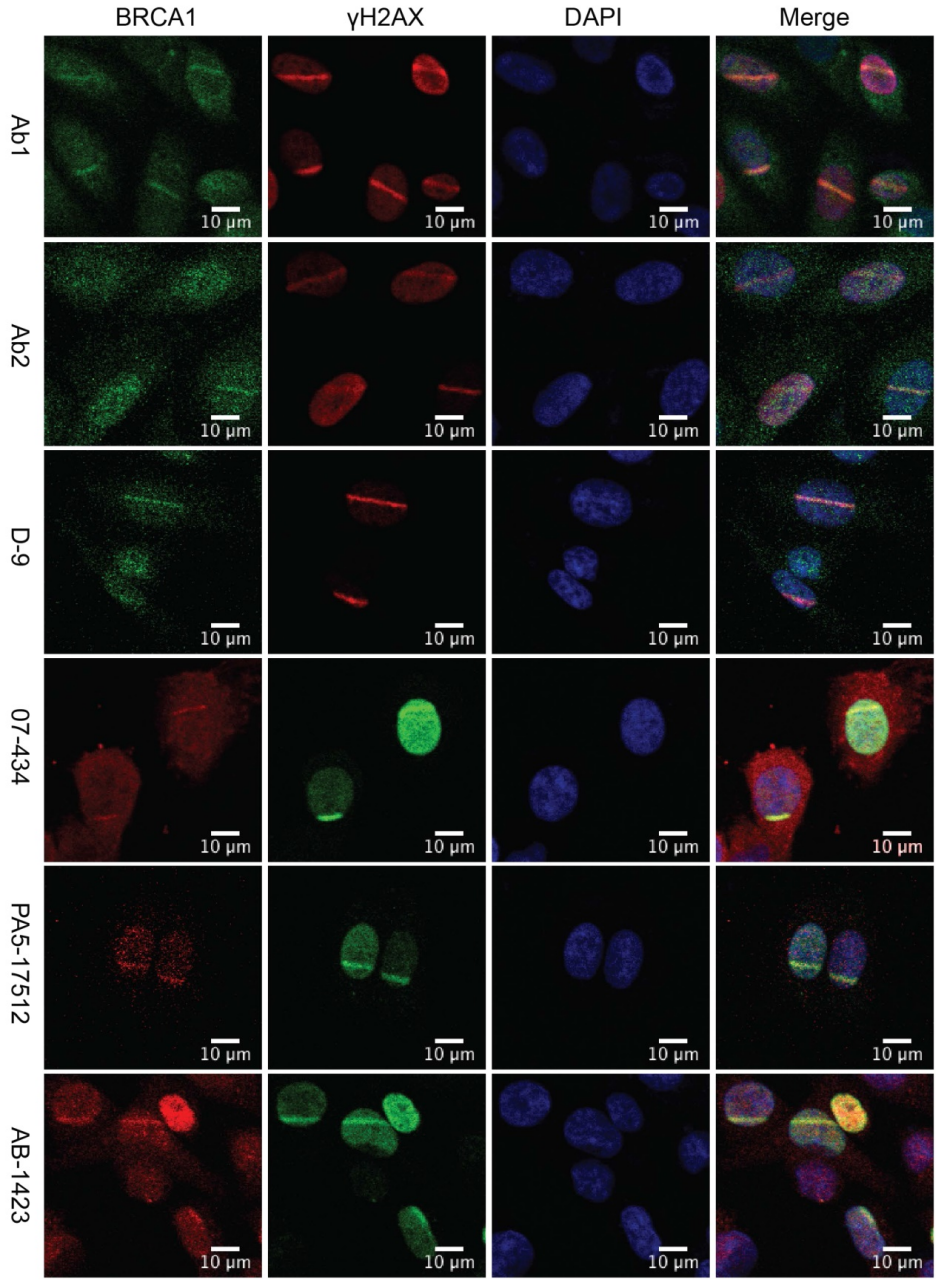
** Analysis of BRCA1 antibodies by immunofluorescence in human U2OS cells.** Laser stripes of BRCA1 and γH2AX at 1h time point after laser micro-irradiation in U2OS cells.

U2OS cells are widely used for studying DNA damage response (DDR) 30, 36-38. U2OS cells are the best choice for laser line assay because their morphology (flat and thin) is suitable for laser irradiation. Previous studies revealed that phosphorylation of H2AX by ATM around double strand breaks (DSBs) set off elaborate ubiquitination and SUMOylation cascades to promote recruitment of BRCA1 39, 40. Upon induction of DSBs, BRCA1 is phosphorylated by multiple DNA repair/checkpoint kinases, including ATM, ATR, and Chk2, and recruited to the DNA damage sites 41-44. We examined whether these antibodies could be used to detect the co-localization of BRCA1 and γH2AX at sites of DNA damage by immunofluorescence (IF). U2OS cells were subjected to laser micro-irradiation to generate DSBs in a line pattern. As shown in Figure 3, several antibodies (07-434, Ab1, Ab2, D-9, AB-1423, and # PA5-17512) could detect BRCA1 stripes, which co-localized with γH2AX stripes, after laser micro-irradiation. Of note, PA5-17512 and AB-1423 could not be validated in WB, but did work in IP and IF, probably due to the difference in epitope conformation between native and denatured protein.

### Analysis of BRCA1 antibodies by ChIP-qPCR in human cancer cells

Chromatin Immunoprecipitation (ChIP) is an antibody-based powerful technology to reveal protein-DNA interactions at specific loci or across the whole genome (ChIP-seq). ChIP-qPCR is often performed to analyze proteins binding to a known subset of target regions in the genome. ChIP-qPCR is less costly and more time efficient than ChIP-seq method for validation purpose. Previous reports identified that BRCA1 binds to the promoter regions of *BRCA1* 45, 46, *ATRIP* 47, *EXO1* 47, *MAD2L1* 48, and *PPM1D* 49 by ChIP-qPCR and ChIP-seq.

**Figure 4:**
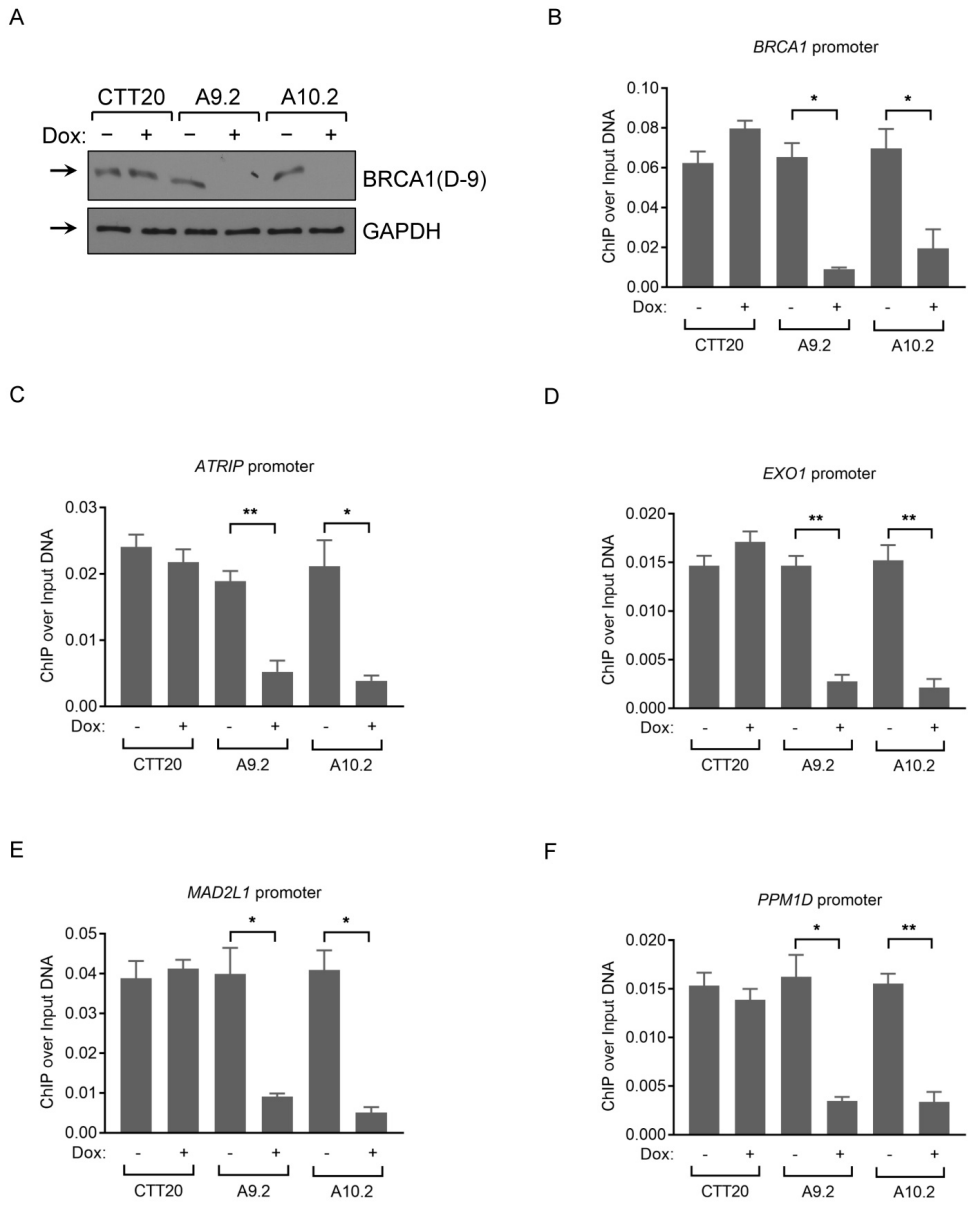
** Analysis of BRCA1 antibodies by ChIP-qPCR in human cancer cells. (A)** BRCA1 immunoblots with CTT20 (inducible Cas9 in HeLa), A9.2 and A10.2 (two BRCA1 inducible KO cell lines) cells with/without doxycycline treatment.** (B-F)** ChIP-qPCR profiles of CTT20 (inducible Cas9 in HeLa), A9.2 and A10.2 cells with/without doxycycline treatment using BRCA1(Ab1) antibody at the *BRCA1* (B), *ATRIP* (C), *EXO1* (D), *MAD2L1* (E) and *PPM1D* (F) promoter regions. 6 μg of Ab (if concentration of Ab is provided) or 15 μl of serum (if concentration is not provided) is used. **p < 0.01, *p < 0.05, Student's t-test.

We chose the following BRCA1 binding promoter regions: *BRCA1*, *ATRIP*, *EXO1*, *MAD2L1*, *PPM1D*, for validation purpose using 12 BRCA1 antibodies based on published BRCA1 and RNA pol II ChIP-seq datasets from Hela, HepG2 and U2OS cell lines (Figure S1). We performed three independent ChIP experiments in U2OS cells and observed a significant enrichment at these promoter regions with Ab1, Ab5 and A300 antibodies (Figure S2A-E), confirming published results. Ab1 gives the highest binding affinity among all 5 loci tested, and RNA pol II binding results were included as positive controls (Figure S2A-E). Of note, A300 antibody did not detect BRCA1 protein convincingly in WB, as the intensity of the band did not change in siRNA-mediated BRCA1 knockdown samples, but did seem to recognize BRCA1 in IP. To examine whether BRCA1 ChIP signal obtained by Ab1 antibody is BRCA1-dependent, we used inducible Cas9 Hela control (CTT20) and BRCA1 KO (A9.2 and A10.2) cell lines in the ChIP assay (Fig. 4A). We first confirmed that BRCA1 associates with the promoter regions of *BRCA1*, *ATRIP*, *EXO1*, *MAD2L1*, and* PPM1D* in HeLa cells expressing full-length BRCA1. Importantly, BRCA1 depletion resulted in loss of BRCA1 recruitment to DNA, compared with control CTT20 cells treated with/without doxycycline (Fig. 4B-F). These results demonstrate that BRCA1 ChIP signals are in a BRCA1-dependent manner, as deletion of BRCA1 significantly attenuates the binding signals to these loci. Ab1 (MS110) antibody gave us the highest binding affinity among the tested antibodies in ChIP assay and this antibody was previously used in ChIP assay by various labs 48-51.

### Validation of BRCA1 antibodies in Western blot with mouse testis and MEF cells

The lack of commercially available BRCA1 antibodies with the specificity required for cell biology assays is a major obstacle in functional analysis of mouse BRCA1 protein 52. Most commercial BRCA1 antibodies were raised against human BRCA1. It was not clear how many of them could recognize mouse BRCA1 protein. Initially we used siRNA to knockdown BRCA1 in immortalized mouse embryonic fibroblast (MEF) cells in order to validate antibody specificity for mouse BRCA1. We found that several antibodies could detect a signal at expected position, but the signal did not change when BRCA1 was knocked down. This could be explained by two possibilities. One is that these antibodies do not recognize BRCA1 and the signal detected is from cross reaction of other proteins (nonspecific band). The other possibility is that protein level and mRNA level may not always be in sync in a snap shot at the time of cell harvest. In other words, although mRNA level is significantly down in siRNA treated cells, the protein level may not reduce significantly at the time of cell harvest. The second possibility is less likely, but cannot be excluded entirely. We therefore decided to use BRCA1 knockout mouse as a negative control in our antibody validation analysis. Earlier report showed that Brca1^Δ11/Δ11^ embryos died at embryonic days 12-18, and haploid loss of p53 could fully rescue Brca1^Δ11/Δ11^ embryonic lethality phenotype 53, 54. We initially obtained p53^+/-^; Brca1Co^Co/Co^; MMTV-Cre mouse strain (in which Brca1 exon 11 is floxed and knocked out in mammary gland in the presence of MMTV-Cre, a gift from Dr. Furth at Georgetown University 32) for other purposes. In the breeding process, we occasionally got a few whole-body Brca1^Δ11/Δ11^ (i.e. p53+/-; Brca1^Δ11/Δ11^) mice in addition to mammary gland-specific Brca1 KO mice, due to MMTV-Cre leakage in oocytes, which has been observed and reported by other labs previously 55.

**Figure 5:**
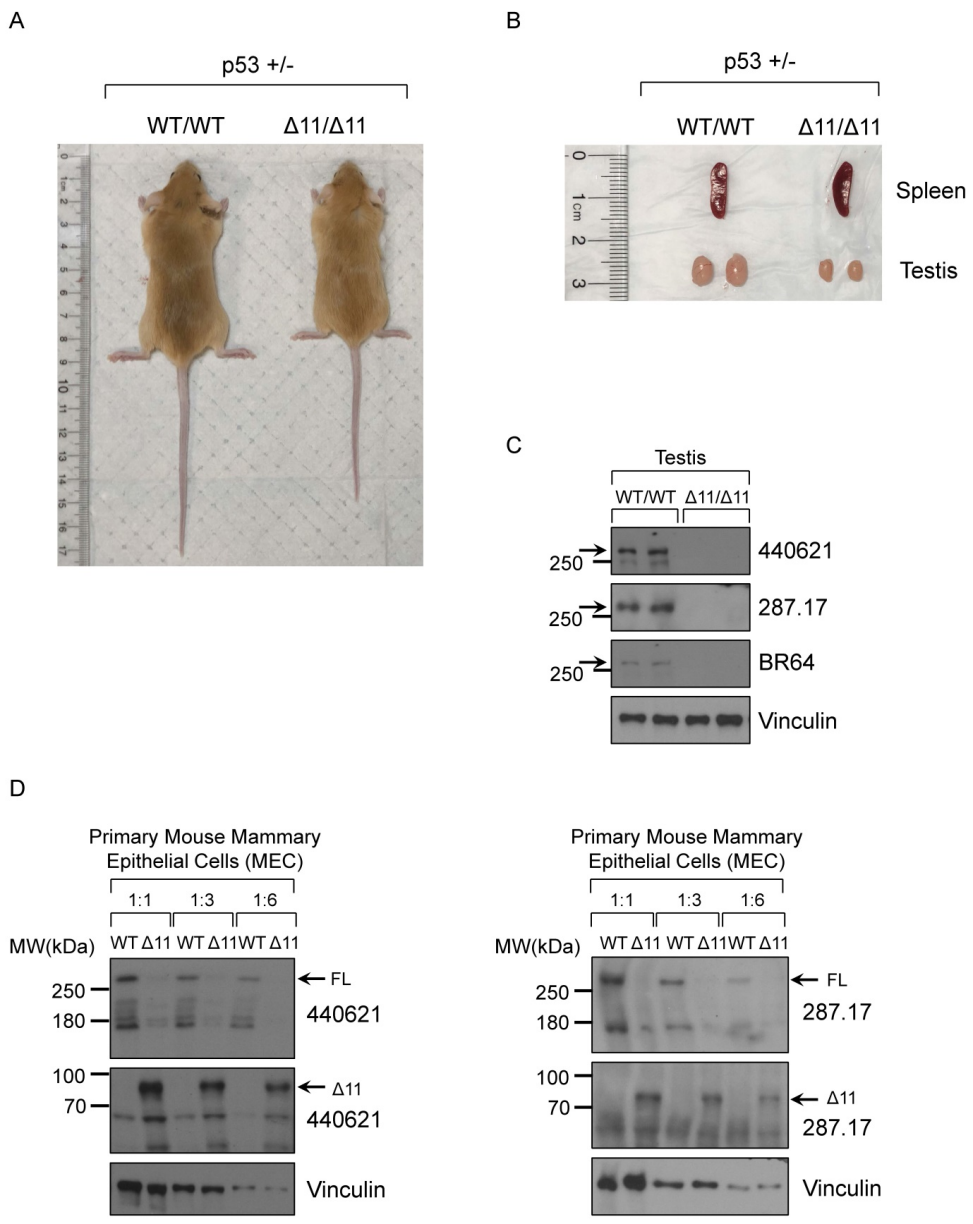
** Validation of BRCA1 antibodies in Western blot with mouse testis and MEC cells. (A)** A representative 8-week-old BRCA1^Δ11/Δ11^ mouse on the right was smaller compared with its WT littermate on the left in the p53+/- background. **(B)** The spleen and testis isolated from BRCA1^Δ11/Δ11^ and WT littermate male mice at 2-month old. **(C)** BRCA1 WB with testis tissue lysate from BRCA1^Δ11/Δ11^ and WT littermate male mice at 2-month old. **(D)** BRCA1 WB with primary mouse mammary epithelial cell lysate from BRCA1^Δ11/Δ11^ and WT littermate female mice at 6-week old.

Consistent with previous findings, two-month old male Brca1^Δ11/Δ11^ mice (whole body KO) have smaller body size (Fig. 5A) and testis (Fig. 5B), compared to WT (wild type) littermate mice in the background of p53^+/-^ 56, 57. Based on others reports and ENCODE RNA-seq results, mouse BRCA1 mRNA and protein are highly expressed in ovary and testis tissues. Due to limited materials for further analysis, here we chose the mouse testis tissue in Western blot assay. After screening 19 antibodies we found that three mouse monoclonal antibodies, BR64, 287.17 and # 440621, could detect full-length mouse BRCA1 protein in testis tissue lysates only from WT mice, but not from Brca1^Δ11/Δ11^ mice (Fig. 5C). Similarly, with cell lysates from cultured primary mouse mammary epithelial cells (MEC), we also detected the full-length BRCA1 protein in wild type cells but disappeared in Brca1^Δ11/Δ11^ MEC samples using 287.17 and # 440621 antibodies (Fig. 5D). In contrast to the full-length BRCA1 protein, BRCA1-Δ11 58 isoform expression significantly increased in primary MEC derived from Brca1^Δ11/Δ11^ mice. We were not able to detect the full-length mouse BRCA1 protein in WT MEC samples using BR64 antibody. This could be due to lower BRCA1 expression in mouse mammary gland compared with that in testis, which is consistent with mRNA expression level in various mouse tissues 17. In addition, BR64 antibody appears to have lower binding affinity for mouse BRCA1 compared to 287.17 and #440621 antibodies.

### Validation of BRCA1 antibodies in MEF cells

In addition to tissue and primary cells derived from p53^+/-^;Brca1^+/+^ and p53^+/-^; Brca1^Δ11/Δ11^ mice, we also wanted to verify antibody specificity with MEF cells, as MEF cells are considered to be more homogeneous and consistent from experiment to experiment. P53^+/-^; Brca1^+/Δ11^ mice were inbred to isolate WT and Brca1^Δ11/Δ11^ littermate MEF. Although live birth of Brca1^Δ11/Δ11^ pups in the background of p53^+/-^ is very low, we found that the ratio of Brca1^+/+^:Brca1^+/-^: Brca1^Δ11/Δ11^ embryos at the time of MEF isolation is close to 1:2:1. WT and Brca1^Δ11/Δ11^ MEF from individual embryos of same litters were immortalized and their genotypes were confirmed by PCR and sequencing. Crude lysates from these immortalized MEF, WT or Brca1^Δ11/Δ11^, were used in WB analysis using all commercial BRCA1 antibodies. We found that only 287.17 and BR64 antibodies could detect the full-length BRCA1 that disappeared in Brca1^Δ11/Δ11^ MEF cells (Figure S3). 07-434 antibody could detect a strong signal corresponding to the size of the full-length BRCA1. Although that band recognized by 07-434 was consistently reduced in Brca1^Δ11/Δ11^ MEF cells, the band was never completely gone, suggesting that 07-434 might also bind to other proteins with similar size (Figure S3). The remaining antibodies can be divided into two groups: those that do not recognize BRCA1 at all or those that recognize bands around expected position but the intensity of these bands does not change in Brca1^Δ11/Δ11^ cells.

**Figure 6:**
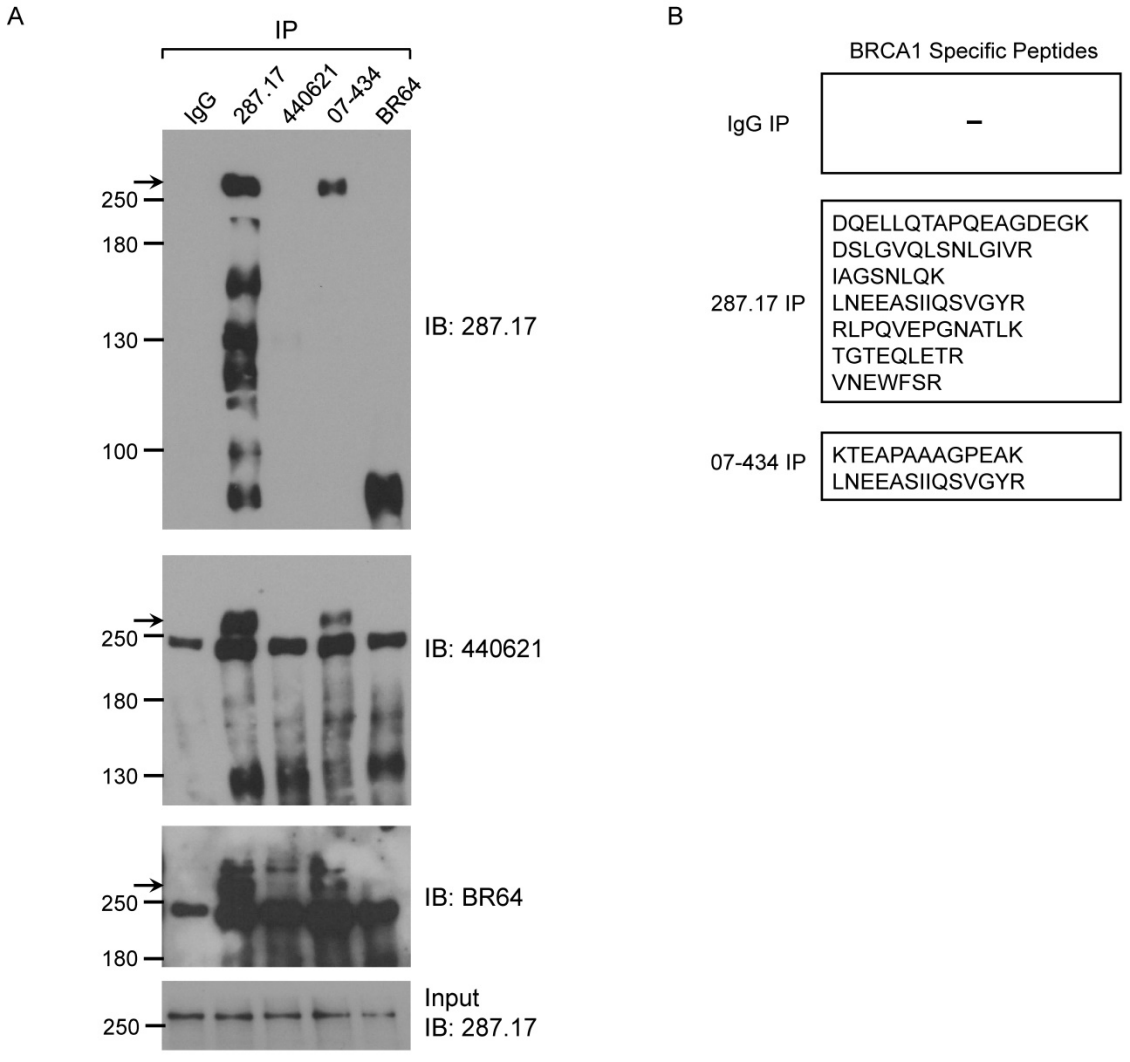
** Validation of BRCA1 antibodies in MEF cells. (A)** MEF cell lysates were prepared and immunoprecipitation was performed using the indicated BRCA1 antibodies pre-coupled to protein A/G beads. **(B)** Summary of BRCA1 specific peptides detected by MS in IP samples with two anti-BRCA1 antibodies.

We further tested whether these antibodies could IP mouse BRCA1. The presence of mouse BRCA1 in the IP samples was detected by WB using BR64, 287.17 and # 440621 antibodies, respectively. Of note, #440621 antibody did not detect BRCA1 in MEF crude lysate, but appeared to work well in MEF IP samples when mouse BRCA1 was greatly enriched/purified. Of the 4 antibodies used in the IP assay, only 287.17 and 07-434 could efficiently pull-down full-length mouse BRCA1 compared to starting material-Input (Fig. 6A). The mouse BRCA1 IP by 287.17 and 07-434 was also independently verified by mass spectrometry analysis of the immunoprecipitated samples: 7 and 2 BRCA1-unique peptides were detected in the IP-MS samples with 287.17 and 07-434 antibodies, respectively (Fig. 6B). In the same experiment, there was no BRCA1 peptide identified by 287.17 and 07-434 IP with Brca1^Δ11/Δ11^; p53^+/-^ MEF cell lysates. We conclude that both 287.17 and 07-434 bind to mouse BRCA1 protein under undenatured condition. It is hard to compare the binding affinity between these two antibodies, as 287.17 is a mouse monoclonal antibody and 07-434 is rabbit serum without affinity purification.

### Analysis of BRCA1 antibodies by immunofluorescence in MEF cells

**Figure 7:**
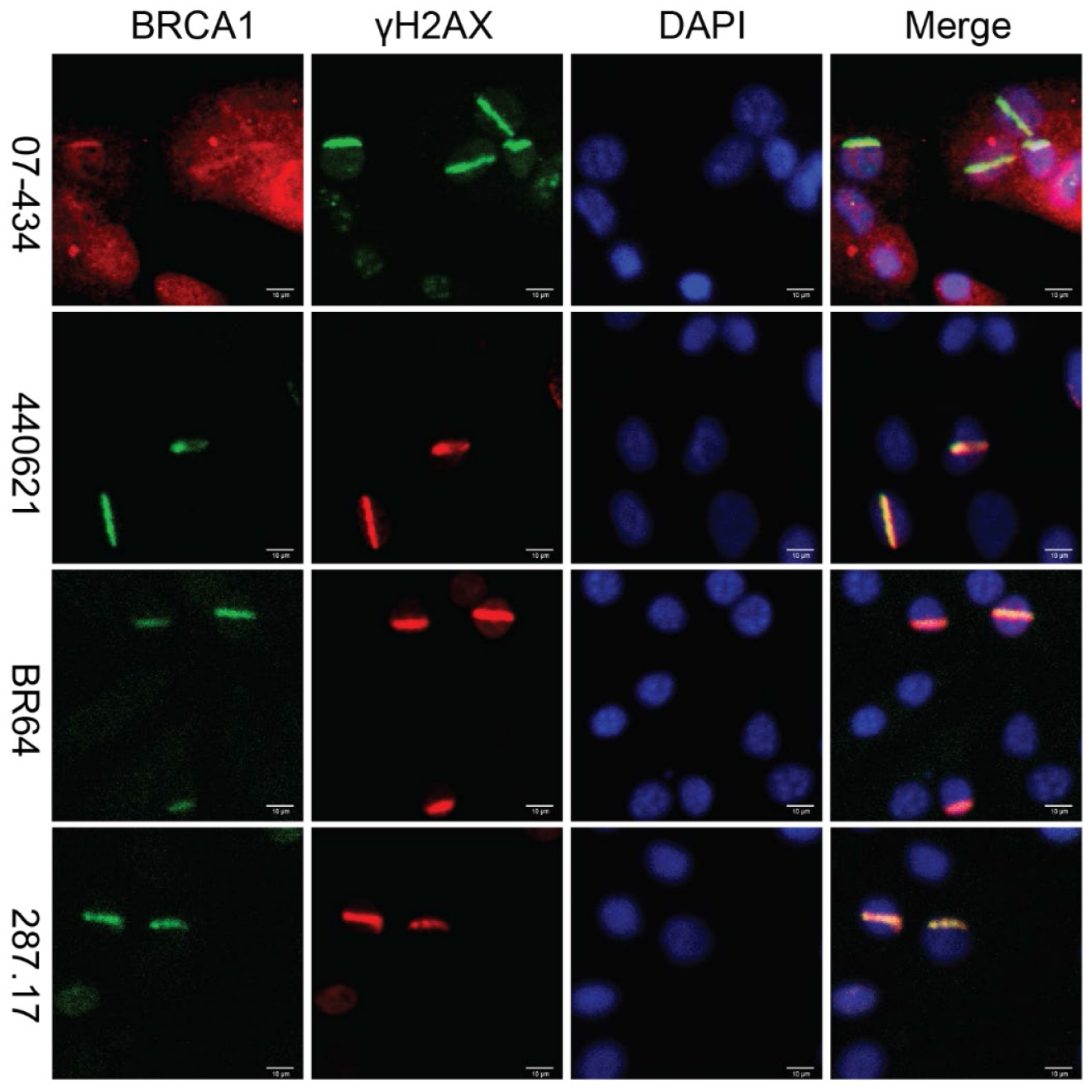
** Analysis of BRCA1 antibodies by immunofluorescence in MEF cells.** Laser stripes of BRCA1 and γH2AX at 1h time point after laser micro-irradiation in MEF cells.

Lastly, we assessed whether these antibodies can be used to detect co-localization of mouse BRCA1 and γH2AX to sites of DNA damage in MEF cells, as immortalized MEF cells are useful tools and have been used in DDR studies. As shown in Figure 7, 07-434, 287.17, #440621 and BR64 could detect BRCA1 stripes that co-localized with γH2AX stripes, after laser micro-irradiation.

## Discussion

A lack of standardized guidelines for determining the specificity and reproducibility of antibodies has caused controversies, generation of dubious data and huge amounts of wasted money 3, 59. Many antibodies became commercially available without careful characterization or proper quality control analysis. Recently, the National Institutes of Health (NIH) and Agency for Healthcare Research and Quality (AHRQ) released new guideline for research grant submission that requires investigators to describe how they will “ensure the identity and validity of key biological and/or chemical resources”, including antibodies (NOT-OD-16-011 and NOT-OD-17-068). In light of this, we sought to test and validate twenty-three commercially available BRCA1 Abs in a systematic manner that addresses these concerns (see Supplemental Table).

The cost of BRCA1 antibodies used in our study can be substantial with the price ranging from approximately $100 to $400 depending on quantities. Thus, investigators mostly rely on company product datasheet, previous publications or other on-line resources (The Human Protein Atlas, https://www.proteinatlas.org; Antibodypedia, https://www.antibodypedia.com) when choosing an antibody 60. At present, there are 4,386,900 reviewed antibodies from 92 Ab vendors, covering gene-products encoded by 19,106 genes (approximately 94% of all human genes) in the community-based database resource Antibodypedia. Here, we highlight the field of BRCA1 as an example of this widespread problem. For example, Rabbit anti-human polyclonal antibody 20649-​1-AP, which has been used in 9 publications, cannot detect full-length human BRCA1 protein by Western blot in our analysis. We communicated with the technical support team of the vendor on this Ab performance. Although we did not receive refund, the antibody was removed from company's sale list later. Currently, the company stated “We are very sorry that this product is still in testing. We will update the datasheet as soon as we get any result” on their product website. The rabbit anti-human polyclonal antibody HPA057371, which was raised against a recombinant protein corresponding to amino acids 1742-1814 of human BRCA1, worked neither for Western blot, nor for IP or IF assays in our study, however, its poor performance was never documented. This kind of unreliable antibodies led to tremendous waste of resources for researchers and questionable data.

Antibodies are often advertised with images of Western blots to indicate their specificity 61. However, one important issue in antibody specificity is cross-reactivity, which is defined as antibodies binding to proteins other than the intended target 62. Only 0.5-5% of the antibodies in a polyclonal reagent bind to their intended target 3, a blot showing a single band at the expected position with estimated masses is not a definitive evidence for Ab specificity. Earlier report found that the commercially available C-20 antibody (discontinued, Santa Cruz) raised against a peptide mapping at the C-terminus of human BRCA1 (1843-1862aa) cross-reacts with human EGFR and HER2 52. Another antibody, I-20, raised against residues 1823-1842 of human BRCA1 (discontinued, Santa Cruz) is still being used in BRCA1 studies, but it didn't work in all the assays under our rigorous conditions, consistent with earlier validation results 63-65. There are several well documented examples of cross-reactive antibodies erroneously used in biomedical research 27. For example, anti-ERβ (Estrogen receptor beta) antibodies NCL-ER-BETA (Leica Biosystems), 9.88 (Millipore Sigma) and 14C8 (GeneTex) cross-react with multiple other proteins 35, 66-68; anti-EpoR (Erythropoietin receptor) antibodies C20 and M20 can detect HSP70 protein as well 69. These unreliable commercial antibodies led to many potentially confusing even incorrect conclusions.

Many commercial antibodies are validated by quick assays where the antigen is completely or partly denatured such as Western blotting or IHC. However, these antibodies may not work in other assays where a protein is in its native conformation and antigen is not exposed, such as in IP and IP-MS 70. There is a need to identify antibodies which recognize antigens in both native and denatured forms 70. Our systematic analysis uncovered several valuable antibodies (287.17, #440621, BR64 and 07-434) that recognize full-length mouse BRCA1 in multiple applications such as WB, IP, IF, but often with their unique complexity. Knowledge of these nuanced complexity could help researchers interpret their data in a more rigorous manner.

Choosing an appropriate antibody for a ChIP assay is vital to its success. Antibodies used in a ChIP experiment should be specific to target protein and have high affinity for the antigen. If validated ChIP-assay quality antibody is not available for the gene of interest, a good starting choice is an antibody that has been validated in IP assay. Since not all IP-validated antibodies work in ChIP and, vice versa, not all ChIP-validated antibodies work in ChIP-seq, it is important to validate antibodies as stringently and thoroughly as possible to ensure data reliability. The more an antibody is validated across other applications, such as WB, IP and IF, the more confidence one might have on antibody specificity and selectivity.

BRCA1 has been depleted in mouse by various tissue-specific Cre recombinase expressing lines, including mammary gland 71, T cells 72, brain 73, 74, heart 18, bone marrow 21 and bone 75, thus the identification of high-quality antibodies for mouse BRCA1 would be important for further study of the functions of BRCA1 at cellular level. The inability to convincingly detect mouse BRCA1 protein is likely one of the reasons for lack of functional studies in various BRCA1 animal models. Use of unspecific or unreliable antibodies for mouse endogenous BRCA1 protein detection occurred in the field in the past three decades. In this study, we found that antibody 287.17 specifically recognizes mouse BRCA1 protein in WB, IP and IF, consistent with reports from recent publications 76, 77. We hope our rigorous characterization of BRCA1 antibodies will be helpful to researchers in the BRCA1 field especially those who study BRCA1 in mouse models. The mouse *BRCA1* gene is composed of 24 exons. Exon 11 is the largest exon among 24 exons and encompasses around 60% of the coding *BRCA1* sequence, containing two nuclear localization sequences (NLS) 71. The exon11 encoded domain interacts with RAD51 43, the RAD50 complex 78, FANCA 79 and p53 80. Moreover, several serine/threonine residues, which are phosphorylated by Chk2 44, ATM 42 and ATR 81 in response to DNA damage, lie within exon11. A note of caution: results from Brca1^Δ11/Δ11^ animals may not be solely due to loss of the full-length BRCA1 expression. One cannot exclude the possibility that increased Brca1-Δ11 in-frame isoform expression could potentially contribute to some of the phenotypes 58.

At the time of this manuscript writing, 42 vendors offer a total of 2224 BRCA1 antibodies in the market according to Antibodypedia website. In conclusion, we suggest to use 287.17 for mouse BRCA1 proteins in WB, IP and IF assays. One limitation of the present study is that it is not feasible to test all the assays with various conditions for each antibody. Our hope is that our extensive characterization of BRCA1 antibodies with proper controls will provide useful information to the scientific community when reviewing past reports and choosing the right antibody for their corresponding assays in the future studies 82.

## Supplementary Material

Supplementary figures.

Supplementary table.

## Figures and Tables

**Table 1:** Antibodies used in this study

Name	Company	Catalog Number
BRCA1	Cell signaling	#14823
BRCA1	Cell signaling	#9010
BRCA1	Millipore Sigma	07-434
BRCA1	Sigma-Aldrich	HPA057371
BRCA1 (AB-1423)	Sigma-Aldrich	SAB4300490
BRCA1	Thermo Fisher	# PA5-17512
BRCA1	Bethyl Laboratories	A300-000A
BRCA1 (I-20)	Santa Cruz	sc-646
BRCA1 (H-300)	Santa Cruz	sc-28234
BRCA1	Proteintech	20649-1-AP
BRCA1 (MS110, Ab1)	Millipore Sigma	OP92
BRCA1 (MS13, Ab2)	Millipore Sigma	OP93
BRCA1 (SG-11, Ab3)	Calbiochem	OP94
BRCA1 (SD118, Ab4)	Millipore Sigma	OP107
BRCA1 (Ab5)	Oncogene Science	AP16
BRCA1 (BR64)	Millipore Sigma	MAB4132
BRCA1 (D-9)	Santa Cruz	sc-6954
BRCA1 (6B4)	Thermo Fisher	# MA1-23164
BRCA1 (17F8)	Thermo Fisher	# MA1-23160
BRCA1 (8F7)	Thermo Fisher	# MA1-23162
BRCA1 (#440621)	R&D Systems	MAB22101
BRCA1 (287.17)	Santa Cruz	sc-135732
BRCA1 (G4)	Santa Cruz	sc-514640
Tubulin	Sigma-Aldrich	CP06
GAPDH	Cell signaling	#2118
Vinculin	Proteintech	66305-1-Ig
γH2AX	Thermo Fisher	05-636
γH2AX	Cell Signaling	9718S
IgG Donkey anti-Rabbit, Alexa Fluor 488	Thermo Fisher	A21206
IgG Donkey anti-Mouse, Alexa Fluor 546	Thermo Fisher	A10036
