# Supplementary material for: BRCA1 Antibodies Matter
Source: Int J Biol Sci. 2021 Jul 25;17(12):3239–54. doi: 10.7150/ijbs.63115 (PMC8375228; doi:10.7150/ijbs.63115)
Supplement: Supplementary file 1 — Supplementary figures. [file ijbsv17p3239s1.pdf]

Supplemental Figure 1. BRCA1 and pol II binding regions by public ChIP-seq datasets.

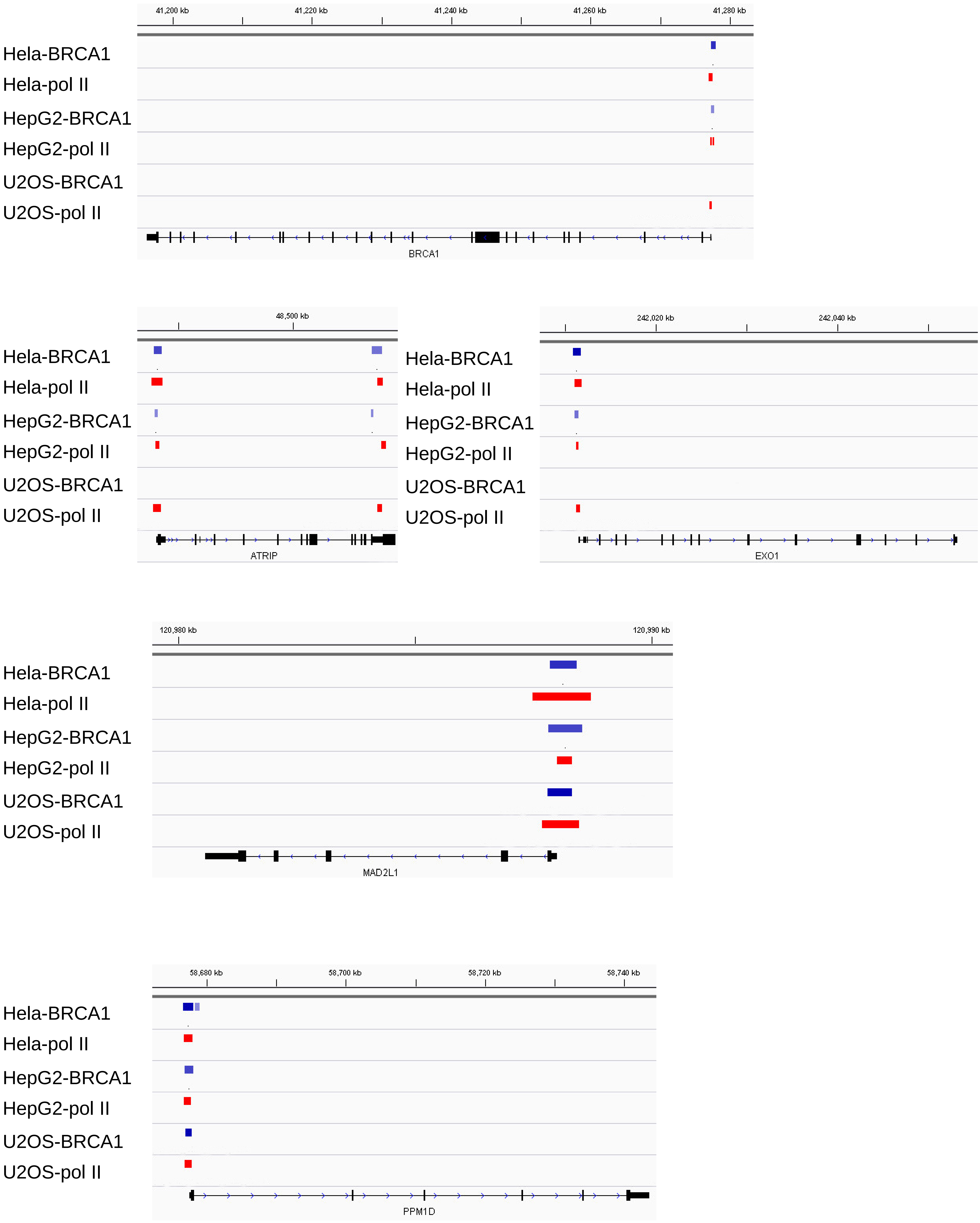

BRCA1 and Pol II ChIP-seq profiles from public datasets at the BRCA1 (A), ATRIP (B), EXO1 (C), MAD2L1 (D) and PPM1D (E) promoter regions.

Supplemental Figure 2. Analysis of BRCA1 antibodies by ChIP-qPCR in human cancer cells.

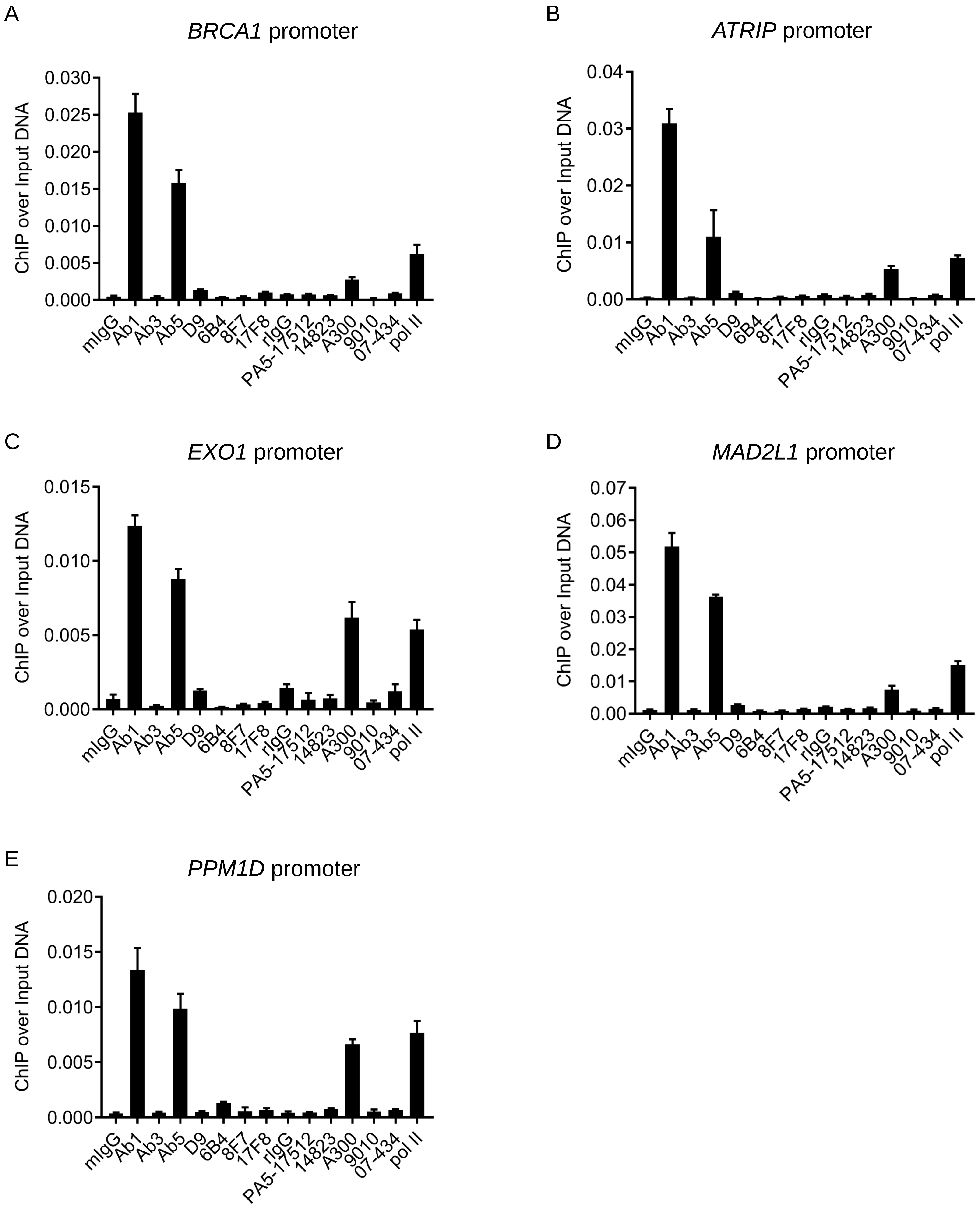

ChIP-qPCR profiles of U2OS cells using different BRCA1 antibodies at the BRCA1 (A), ATRIP (B), EXO1 (C), MAD2L1 (D) and PPM1D (E) promoter regions. 6 ug of Ab (when Ab concentration is known) or 15 ul of serum (when Ab concentration is not provided) is used in each ChIP. Only 2 ug of PolII Ab is used.

Supplemental Figure 3. Validation of BRCA1 antibodies in western blot application with MEF cells.

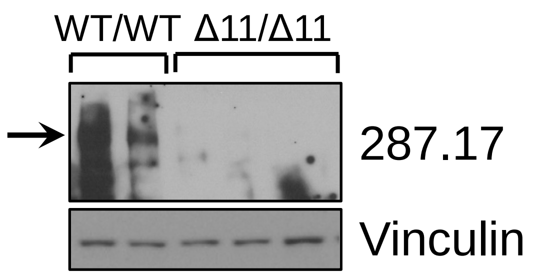

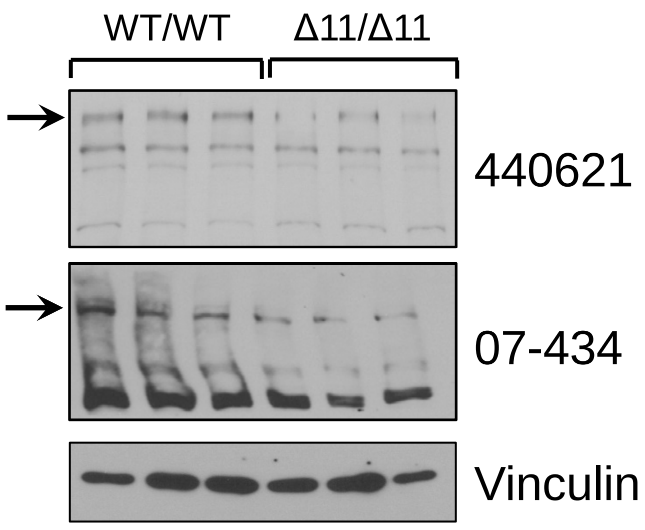

BRCA1 Abs test with MEF WT and/or BRCA1 Δ11/ Δ11 cells.
